# Ethnicity, gender and community sentences

**DOI:** 10.23889/ijpds.v8i2.2300

**Published:** 2023-09-14

**Authors:** Angela Sorsby

**Affiliations:** 1 University of Bristol, Bristol

## Objectives

This paper presents findings from administrative data analysis examining differences between ethnic groups and men and women in the number and type of requirements that make up community sentences as well as the effectiveness of different requirements in terms of successful completion of the sentence.

## Methods

The paper presents findings from analysis of the Data First probation and criminal justice linked datasets. The analysis will focus on whether:

- there are differences between ethnic groups and men and women in the number and type of requirements that make up community-based orders (rehabilitation, unpaid work, curfew and accredited programmes)
- some requirements are more effective in terms of successful completion of the order.

The paper presents findings from regression analysis used to examine the above relationships while controlling for other relevant variables such as age, number of previous convictions and offence type.

## Results

Graphs will be used to set out differences between broad ethnic groups and men and women in the total number of requirements which make up community-based orders and the proportion of offenders from each group which receive each of the main types of requirement namely: rehabilitation; unpaid work; curfew; and accredited programmes. Graphs will also be used to set out differences between the different order requirements in terms of successful completion. The paper will also present findings from regression analysis which will identify differences after taking account of other factors. The findings will of necessity be based on broad ethnic groups as it is unlikely that there will be sufficient numbers of people within more narrow ethnic groups to meet statistical disclosure criteria.

## Conclusion

There is a lack of information on relationships between ethnicity, gender and community sentences. Better understanding of these relationships has been identified as crucial by HM Inspectorate of Probation. This paper provides more information on these relationships enabling policy decisions to be better targeted to provide equality of outcomes.

